# Recalcitrant Lateral Premalleolar Bursitis of the Ankle Associated with Lateral Ankle Instability

**DOI:** 10.1155/2017/4854812

**Published:** 2017-08-03

**Authors:** Masashi Naito, Takumi Matsumoto, Song Ho Chang, Masachika Ikegami, Jun Hirose, Sakae Tanaka

**Affiliations:** Department of Orthopaedic Surgery, Faculty of Medicine, The University of Tokyo, 7-3-1 Hongo, Bunkyo-ku, Tokyo 113-8655, Japan

## Abstract

Lateral premalleolar bursitis of the ankle is a rarely reported disorder in the English literature although it is not uncommon in Asian countries where people commonly sit on their feet. Here, we present the case of a 66-year-old woman with recalcitrant lateral premalleolar bursitis associated with lateral ankle instability which was successfully treated with surgical resection of the bursa and repair of the anterior talofibular ligament. Operative findings revealed a communication between the bursa and articular cavity of the ankle joint via the sheath of the extensor digitorum longus tendon, which was considered to act as a check valve leading to a large and recalcitrant bursitis. This report provides a novel concept about the etiology of recalcitrant lateral premalleolar bursitis of the ankle.

## 1. Introduction

A bursa is a cyst lined with synovial cells and located in an area exposed to high pressure or repetitive friction. Bursitis is the inflammation of the bursa mainly caused by excessive mechanical stimulation and by other reasons including autoimmune inflammatory diseases, trauma, and infection. Some bursae located adjacent to joints may have communication with joints. Such bursae are termed as communicating bursae and have been reported to occur around the hip, knee, and shoulder joints [1–3]; however, there is no report in the English literature regarding communicating bursae around the ankle joint.

The foot and ankle region is one of the commonest sites of bursitis because it is subject to mechanical stress from the external environment. Lateral premalleolar bursitis, known as an occupational bursa among floor layers in Western countries, is not uncommon among the general population in Asian countries where sitting on the foot is popular [4]. Most cases of bursitis are managed conservatively with methods such as local protection against stimuli, aspiration, a compressive wrap, and corticosteroid injection [5]. Operative excision is performed for recurrent and symptomatic cases unresponsive to conservative treatments.

Here we report a case of repetitive lateral premalleolar bursitis resistant to conservative treatment, which was revealed as a communicating bursa associated with ankle instability.

## 2. Case Report

**Figure 1:**
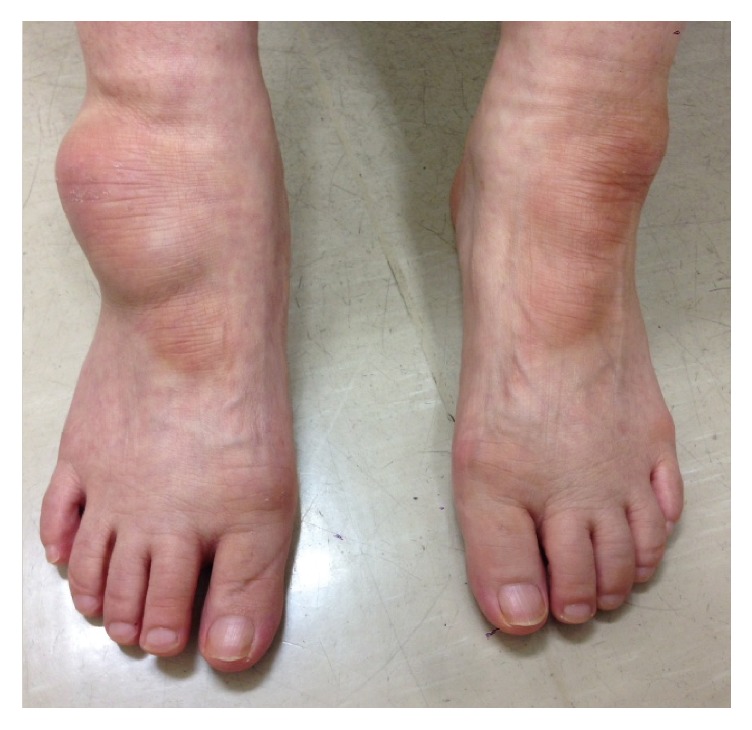
The appearance of the ankles before surgery. The anterolateral area of the right ankle was swollen and had an overlying callus. A callus may also be seen in the same position in the left foot.

A 66-year-old woman complained of an intractable swelling of the right ankle and difficulty in wearing shoes on the affected side because of the swelling. She had a medical history of type 2 diabetes, hypertension, and dyslipidemia. The patient had become blind due to diabetic retinopathy at the age of 35 years. After an inversion sprain of her right ankle which occurred two years previously, which was treated conservatively by her local doctor, she began to feel discomfort in the ankle. She noticed that the anterolateral part of the ankle gradually got swollen. The patient visited a nearby orthopedic clinic about a year after the episode of ankle sprain and was diagnosed with lateral premalleolar bursitis. Conservative treatment including several aspirations and corticosteroid injections failed to reduce the size of the bursitis, and the patient was referred to our hospital for surgical treatment. Physical examination revealed a fluctuant mass 5 × 8 cm in size, over the anterolateral part of the right ankle (Figure 1). There was no local heat or redness. A callus was formed just over the surface of the mass on the right foot and at the same position on the left foot. Tenderness was localized around the anterior talofibular ligament (ATFL), and instability and apprehension were evoked by the anterior drawer test. Clear yellowish fluid was aspirated from the mass and was cultured, but no organism growth was observed.

**Figure 2:**
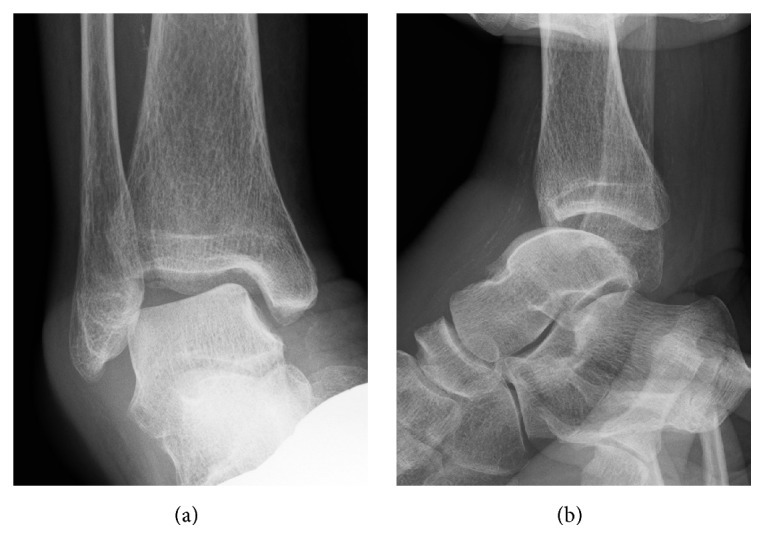
(a) Varus stress view of the ankle showing the talar tilt of 15 degrees. (b) Anterior drawer stress view of the ankle showing marked anterior displacement of the talus.

**Figure 3:**
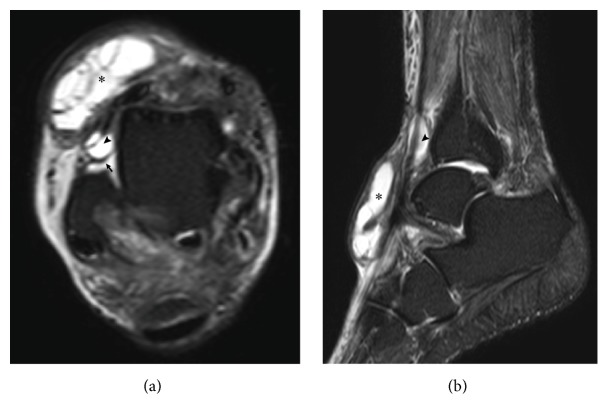
Magnetic resonance imaging of the right ankle. (a) An axial short T1 inversion recovery (STIR) image showing fluid collection in the lateral premalleolar bursa (asterisk). Fluid collection may also be seen in the tendon sheath of the extensor digitorum longus (EDL) (arrowhead). The anterior talofibular ligament is unstrained (arrow). (b) A sagittal STIR image showing fluid collection in the tendon sheath of the EDL just anterior to the ankle joint.

Plain radiographs revealed no apparent abnormality except for a round soft tissue shadow corresponding to the lesion in the anterolateral aspect of the ankle. Varus and anterior instability were obvious with stress radiography (Figure 2). Magnetic resonance imaging (MRI) of the right ankle displayed a homogeneous multicystic lesion in contact with the anterolateral capsule of the ankle joint that had isointensity on T1-weighted, high intensity on T2-weighted, and high intensity on short T1 inversion recovery (STIR) images, indicating fluid collection in the lateral premalleolar bursa. MRI also showed unstrained ATFL and fluid collection in the sheath of the extensor digitorum longus (EDL) tendon (Figure 3). These clinical findings and her past history prompted us to consider that chronic ankle instability and the disrupted capsule due to the past ankle sprain might have contributed to the recalcitrant bursitis with the disrupted capsule working as a check valve.

**Figure 4:**
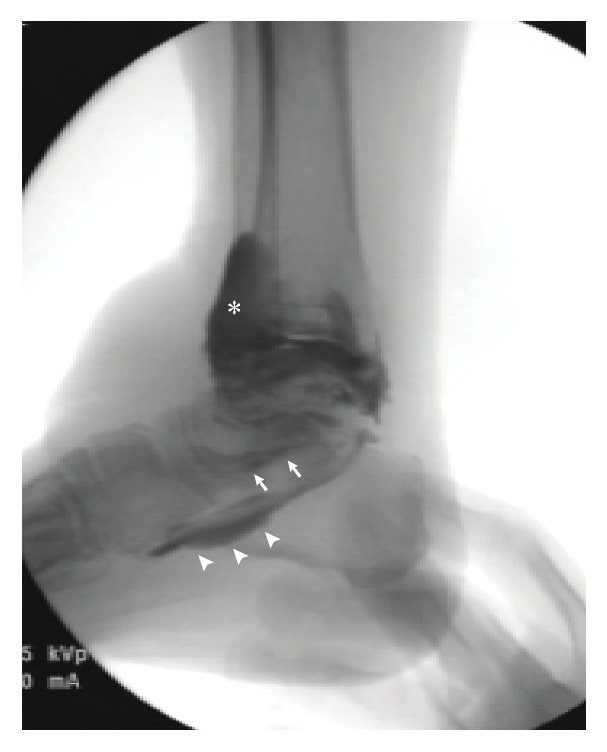
Arthrogram of the right ankle. Constant medium leaked anteriorly into the syndesmosis and the tendon sheath of the extensor digitorum longus (asterisk). Leakage may also be seen in the tendon sheaths of the tibialis posterior (arrow) and the flexor hallucis longus (arrowhead).

**Figure 5:**
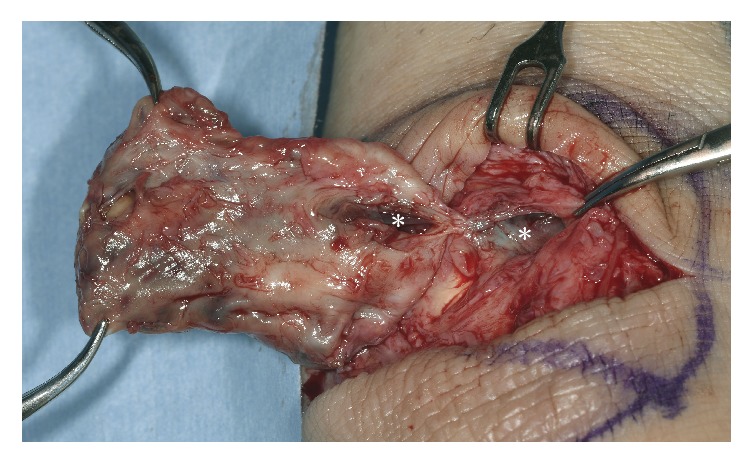
An intraoperative photograph of the resected bursa. The bursal cavity was in communication with the tendon sheath of the extensor digitorum longus (asterisks).

We performed bursectomy and ATFL repair for this case. In the operating room, ankle arthrography was performed before the surgery. Contrast medium was injected into the right ankle joint and the joint was passively moved to spread the medium. The arthrogram showed contrast medium leaking from the anterior aspect of the joint into the tendon sheath of the EDL and the distal tibiofibular syndesmosis and from the posterior aspect of the joint into the tendon sheath of the flexor hallucis longus (FHL) and the tibialis posterior indicating ruptures of the joint capsule of the ankle joint (Figure 4). No leakage into the bursa was observed. Following the arthrography, indocyanine green was injected into the lateral premalleolar bursa percutaneously so that the margin of the bursa to be resected could be easily visualized. The indocyanine green injection was performed under arthroscopic observation of the ankle joint; however, leakage of the indocyanine green into the joint was not observed. Arthroscopy also revealed the tear of ATFL on the fibular side and a disrupted posterior capsule leading to exposure of the FHL tendon. Next, we performed open bursectomy with a transverse skin incision just above the bursa. The thin cutaneous layer around the bursa was stripped off with particular attention to the dorsal cutaneous nerve. When the underside surface of the bursa was being dissected from the underlying extensor retinaculum, the bursa was found to communicate with the tendon sheath of the EDL through a fistula 5 mm in diameter (Figure 5). The fistula and inner side of the tendon sheath were stained with indocyanine green confirming a communication between the bursa and the tendon sheath. The bursa was completely removed and the fistula was closed with a few absorbable sutures. We then repaired the ATFL according to the modified Broström-Gould method using suture anchors. Postoperatively, full weight bearing was allowed immediately with a soft ankle brace. Activity without a brace was allowed at 12 weeks after the surgery. The surgical wound healed without complications. Histological investigation of the resected specimen revealed hyalinized fibrous tissue with proliferation of microvessels and granulation tissue and the migration of inflammatory cells, compatible with chronic bursitis. The patient was followed up for 17 months without recurrence of bursitis. The patient was satisfied with the result and had no difficulty wearing shoes and no functional disability at the latest follow-up

## 3. Discussion

A bursa is a cyst lined with synovial cells and is usually located over a bony prominence to reduce friction with subcutaneous tissues or tendons [6]. Bursae are divided into two types by etiology. Anatomical bursae develop normally during the process of growth. In contrast, adventitious bursae develop in response to excessive friction [7]. Because the foot and ankle must bear weight and receive mechanical stress from the ground and are exposed to chronic stimulation by socks and shoes, the foot and ankle region is one of the commonest sites where an adventitious bursa may develop [8].

Lateral premalleolar bursitis develops on the dorsolateral part of the foot anterior to the lateral malleolus, which is distinct from lateral malleolar bursitis located just above the lateral malleolus. Lateral malleolar bursitis has been reported in miners sitting with their legs crossed in tunnels with low ceilings [9] or figure skaters whose malleoli are subject to abnormal contact pressure and shear forces from their boots [10]. On the other hand, lateral premalleolar bursitis was first reported by Robertson and Haywood in 1983 as an occupational bursitis among floor layers who sit on their feet during work [4]. Another study from Turkey involving 21 cases of lateral premalleolar bursitis reported that all the patients regularly sat on the floor with their feet under the buttocks during prayer or rest [5]. These epidemiological findings suggest that the major cause of lateral premalleolar bursitis is the repetitive compression and friction between the talar head and floor. In our case, the patient preferred the traditional Japanese lifestyle of sitting on the floor and calluses were observed on the dorsolateral part of both feet.

Bursae can be divided into two categories regarding the presence or absence of communication with the adjacent joint, communicating or noncommunicating bursa. Some communicating bursae become enlarged and recalcitrant because of the communicating tunnel working as a check valve. A popliteal cyst, which is a distension of the bursa existing between the gastrocnemius and the semimembranosus muscles, is a representative of this type of bursa developing as a result of the check-valve mechanism [2, 11]. Articular fluid fills the bursa, and the flow is one-way from the articular cavity to the bursal cavity. In adults, almost all popliteal cysts are related to the pathological state of the knee such as meniscal tears and degenerative arthrosis. A study using MRI reported anterior cruciate ligament insufficiency in approximately 30% of knees with a popliteal cyst [12], suggesting that joint instability results in a one-way valve mechanism and distention of the bursa. In the present case, we found a communication between the lateral premalleolar bursa and articular cavity through the tendon sheath of the EDL in an ankle with instability due to ATFL tear. The patient's history suggests that the onset of bursitis was related to ankle sprain and indicates that the ankle instability was the cause of the bursitis. To our knowledge, no study has reported lateral premalleolar bursitis exacerbated by the check-valve mechanism. In the present case, leakage from the tendon sheath of EDL into the bursa was not observed via arthrography despite the fistula between them. We believe that this might be attributed to our method of spreading the contrast medium which consisted of only passive dorsiflexion and plantar flexion of the ankle. Moreover, the check valve might have been so tight that the unstable ankle with ATFL tear could not produce enough intraarticular pressure to push the contrast medium into the bursa, and the other ruptured sites such as posterior capsule might have been more predisposed to receive the inflow of the medium. We should attempt the passive movement of the ankle in the anterior and posterior direction and lesser toes in extension and flexion in order to activate the check-valve mechanism.

In summary, we reported a rare case of lateral premalleolar bursitis triggered by an ankle sprain. Underlying check-valve mechanism and ankle pathology should be suspected in recalcitrant lateral premalleolar bursitis.
